# Effects of 8 weeks of bed rest with or without resistance exercise intervention on the volume of the muscle tissue and the adipose tissues of the thigh

**DOI:** 10.14814/phy2.14560

**Published:** 2020-09-19

**Authors:** Madoka Ogawa, Daniel L. Belavý, Akito Yoshiko, Gabriele Armbrecht, Tanja Miokovic, Dieter Felsenberg, Hiroshi Akima

**Affiliations:** ^1^ Graduate School of Education and Human Development Nagoya University Furo Nagoya Aichi Japan; ^2^ Charité‐Universitätsmedizin Berlin Freie Universität Berlin Humboldt‐Universitätzu Berlin Berlin Institute of Health Institute of Radiology Berlin Germany; ^3^ School of International Liberal Studies Chukyo University Toyota Japan; ^4^ Research Center of Health Physical Fitness and Sports Nagoya University Furo‐cho Nagoya Japan

**Keywords:** disuse, intermuscular adipose tissue, intramuscular fat, muscle atrophy, resistance training, unloading model

## Abstract

The present study aims to investigate the effects of 8 weeks of bed rest, with or without resistance exercise intervention, on the volumes of muscle tissue and the intramuscular, intermuscular, and subcutaneous adipose tissues of the thigh. Twenty men were included, who were randomly assigned in three groups: resistance exercises group (RE group), resistance exercises with whole‐body vibration group (VRE group), and nonexercise control group (CTR group). The RE and VRE groups performed resistance exercises during 8 weeks of bed rest (3 days per week). Additionally, consecutive axial magnetic resonance images were obtained before and after the bed rest. Using these images, the volumes of the muscle tissue and the intramuscular adipose tissue, intermuscular adipose tissue, and subcutaneous adipose tissue of the thigh were evaluated. No significant time‐by‐group interaction was observed the volumes of the muscle tissue and the intramuscular adipose tissue, intermuscular adipose tissue, and subcutaneous adipose tissue between the RE and VRE groups. Furthermore, the RE and VRE groups were pooled as the resistance exercise intervention group (TR group), wherein their thigh muscle tissue volume was observed to be maintained after the bed rest. However, that of the CTR group significantly decreased. Regarding the thigh intramuscular adipose tissue and intermuscular adipose tissue volumes, no significant difference was observed among the CTR and TR groups. Although subcutaneous adipose tissue volume in the CTR group significantly increased after the bed rest, no changes were observed in that of the TR group. Therefore, the results of the present study suggested that within the 8 weeks of bed rest, adipose tissue adaptation differs depending on the location

## Graphical abstract

This study aims to investigate the effects of 8 weeks of bed rest, with or without resistance exercise intervention, on the volumes of muscle tissue, intramuscular adipose tissue, intermuscular adipose tissue and subcutaneous adipose tissue of the thigh. Resistance exercise intervention during 8 weeks of bed rest prevented muscle atrophy and subcutaneous adipose tissue increase in the thigh. Additionally, the changes in the intramuscular adipose tissue and intermuscular adipose tissue volumes were not significantly difference in the control and resistance exercise groups. These results demonstrate that after 8 weeks of bed rest, the adipose tissue’s adaptation differs depending on location.

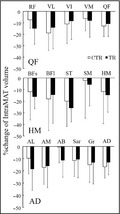

## 1 INTRODUCTION

Many attempts have been made to evaluate the effect of prolonged bed rest (considered as the best physical inactivity model) on human deconditioning, such as muscle atrophy (Akima et al., 2000; Belavý et al., 2009; Pavy‐Le Traon et al., 2007) and adipose tissue accumulation (Belavý et al., 2014; Krebset al., 1990). Some studies have shown that the atrophic pattern induced by bed rest is not uniform among the muscle groups or the individual muscles of the lower limbs (Akima et al., 2000, 2001; Belavý et al., 2010; Miokovic et al., 2014). For example, Belavý et al. (2009) reported that the volumes of the knee extensors, knee flexors, and adductor muscles decreased by 14%, 11%, and 5%, respectively, as a result of 8 weeks of bed rest. They also detailed the atrophic response of the individual muscles on each muscle group in the thigh. Furthermore, the atrophic response in the individual muscles varied, ranged from −15.9% to −5.1%, −12.5% to −7.3%, and 0.8% to −7.0% in the knee extensors, knee flexors, and adductor muscles, respectively. A similar result had been shown in the study of Akima et al. (2000), wherein a variation in the relative volume change of individual muscles in the knee extensors and knee flexors ranged from −8% to −5% and −15% to −5%, respectively, after 20 days of bed rest. Therefore, these previous studies clearly showed the effect of bed rest on the atrophic response among the muscle groups as well as in the individual muscles. Disuse also induces biophysical adaptation in the muscle by an increase in the spin–spin relaxation times (T2) of the skeletal muscle (Akima et al.,  2003, 2008). Increases in the relaxation time of the muscle T2 have been known to indicate increased in that of the intramuscular adipose tissue^2000^ Since this finding is not well supported in the literature, a more detailed image analysis is needed to segment the muscle and adipose tissues.

Adipose tissues are classified depending on their location: (a) subcutaneous adipose tissue (SAT), which is located beneath the skin; (b) intramuscular adipose tissue (IntraMAT), which is located within the muscle; and (c) intermuscular adipose tissue (InterMAT), which is located between the muscles (Shen et al., 2003; Yoshiko et al., 2018). The adipose tissues in the interstitium are referred to as IntraMAT, that is, extramyocellular lipids (Akima et al., 2016), considering the amount of lipids outside the muscle fibers, which is approximately 5‐ to 10‐fold greater than intramyocellular lipids, or that of the lipids within the muscle fibers (Akima et al., 2016). Previous studies reported that the development of muscle atrophy and adipose tissue deposition ultimately results in glucose intolerance and insulin resistance (Elder et al., 2004; Goodpaster et al., 1999). Therefore, quantifying the amount of adipose tissues within or around the muscle are necessary to estimate the impairment of muscle metabolism. However, further research is required on the adipose tissue adaptation as a result of bed rest.

Resistance exercise performed during bed rest has been known to countermeasure muscle deconditioning (Akima et al., 2000, 2001; Alkner & Tesch, 2004; Belavý et al., 2009). For example, Akima et al. (2000, 2001) showed that resistance training performed within 20 days of bed rest attenuates muscle atrophy. Furthermore, resistance exercises combined with whole‐body vibration that were performed during bed rest also have additional training effects. Belavý et al. (2009) and Miokovic et al. (2014) reported that resistance exercises with whole‐body vibration performed during bed rest attenuated muscle atrophy in the lower limbs to the same extent as resistance exercises alone (Belavý et al., 2009; Miokovic et al., 2014). However, the effects of performing resistance exercises with whole‐body vibration or resistance exercise alone on muscle tissue, IntraMAT, and InterMAT need to be further investigated. In cross‐sectional and longitudinal studies, significant negative correlation between muscle tissue size and IntraMAT size were found (Akima et al., 2015; Yoshiko et al., 2018), whereas the link between the changes in muscle tissue size and that in IntraMAT size and InterMAT size during a long‐term longitudinal intervention is unclear.

The present study aims to evaluate the effect of resistance exercises with or without whole‐body vibration performed during 8 weeks of bed rest on the volumes of the muscle tissue, IntraMAT, InterMAT and SAT in the thigh. In the present study, we hypothesized that resistance exercises with or without whole‐body vibration that are performed during bed rest preventmuscle atrophy and the adipose tissue increase.

## 2 MATERIALS AND METHODS

### 2.1 Participants

Twenty‐four healthy men participated in the present study with the compliance of the experimental protocol, which was approved by the ethics committee of the Charité Universitätsmedizin Berlin and that of the Research Centre of Health, Physical Fitness and Sports at Nagoya University. The present study was completed according to the Declaration of Helsinki. The screening ensured that the participants were medically and psychologically healthy. The recruitment process and the bed rest study protocol have been published elsewhere (Belavý et al., 2010). Written informed consent was obtained from all participants before the experiment. Furthermore, each participant was randomly assigned to one of the three groups: resistance exercises group (RE group; *n* = 8), resistance exercises with whole‐body vibration group (VRE group; *n* = 8), or nonexercise control group (CTR group; *n* = 8). Due to the blurred magnetic resonance images, the adipose and muscle tissues of four participants could not be distinguished, and they were excluded from the imaging analysis. Therefore, the groups for the image analysis were classified as follows: RE (*n* = 7), VRE (*n* = 6), and CTR (*n* = 8). The physical characteristics of the participants were shown in Table 1. To reduce interindividual variability, participants who were involved in competitive sports in the past 5 years were excluded from the present study.

**TABLE 1:** Physical characteristics of the participants

	TR group (*n* = 13)		CTR group (*n* = 7)	
	Before	After	Before	After
Age (years)	31.3 ± 7.6	N/A	34.0 ± 7.1	N/A
Height (cm)	179.9 ± 6.3	N/A	181.8 ± 5.6	N/A
Weight (kg)	78.3 ± 10.1	77.7 ± 9.7	81.0 ± 5.8	79.9 ± 5.2
BMI (kg/m^2^)	24.1 ± 1.9	24.0 ± 2.1	24.6 ± 2.5	24.2 ± 2.3

Note

Values are means ± *SD*. BMI, body mass index; CTR, control group; TR, resistance exercise intervention group.

### 2.2 Magnetic resonance imaging protocol

Magnetic resonance (MR) imaging was performed using a 1.5‐T whole‐body scanner (Siemens Avanto, Erlangen, Germany) in two different occasions: 8 or 9 days before and the 55th or 56th day after bed rest. Cushions provided by the MR manufacturer were placed under the knee to support the joint in slight flexion. The MR coil placed over the legs and feet helped to standardize ankle and foot position and ensured that the participant did not need to actively contract their thigh muscles to maintain position. To allow time for the shift of body fluids from the extremities, the participants remained lying horizontally for at least 2 hr before each scanning session. During the bed rest phase, beds were placed in the horizontal position 2 hr before scanning to ensure the comparability of the data before and after bed rest.

Depending on the participant's height, up to 35 axial T1‐weighted images of the greater trochanter to the end of the lateral malleolus were obtained to encompass the left thigh (echo time, 36 ms; repetition time, 5,820 ms; field of view, 450 × 270 mm interpolated to 320 × 192 pixels; slice thickness, 12 mm; interslice distance, 12 mm). The images were stored on a personal computer for later analysis.

### 2.3 Analysis of thigh composition

Medical Image Processing, Analysis and Visualization software (version 4.4.0; National Institutes of Health, Bethesda, MD) was used to analyze the images on a personal computer. This procedure was essentially the same as that in previous studies (Akima et al., 2015, 2016; Yoshiko et al., 2018). The first step of the analysis was to correct for image heterogeneity caused by suboptimal radiofrequency coil uniformity or gradient‐driven eddy currents, using a well‐established nonparametric nonuniform intensity normalization (N3) algorithm (Akima et al., 2015; Manini et al., 2007). This step was essential for subsequent analyses that assumed homogeneous signal intensity across images. Optimized image correction parameters were determined as follows: end tolerance, 0.0001; maximum iterations, 100; signal threshold, 1; field distance, 25 mm; subsampling factor, 4; kernel full width at half maximum, 0.15; and Wiener filter noise, 0.01. The same parameters were applied to all images.

We then calculated the cross‐sectional area (CSA) of the IntraMAT and muscle tissue of the thigh using the previously described threshold method in T1‐weighted imaging. Three regions of interest (ROIs) were isolated in the muscle tissue and SAT (Akima et al., 2015). Next, an auto‐determined threshold was isolated at the base of the first peak of a bimodal histogram. The analysis was repeated three times for each image slice, and the average threshold value was used to classify the tissue pixels. After carefully tracing the edge of each muscle, the following parameters were calculated: (a) the total number of pixels within the ROI, (b) the number of pixels with a signal intensity lower than the threshold value (muscle tissue), and (c) the number of pixels with a value higher than the threshold value. Subsequently, the muscle tissue CSA and IntraMAT CSA were calculated using the following equations:

$$\text{Muscle}\;\text{tissue}\;\text{CSA}\left({\text{cm}}^{2}\right)=\left(\text{muscle}\;\text{tissue}\;\text{pixel}\;\text{number}\right)\times {\left(\text{FOV}/\text{matrix}\;\text{size}\right)}^{2}$$

$$\text{IntraMAT}\;\text{CSA}\left({\text{cm}}^{2}\right)=\left(\text{IntraMAT}\;\text{pixel}\;\text{number}\right)\times {\left(\text{FOV}/\text{matrix}\;\text{size}\right)}^{2},$$

in which FOV is the field of view.

For muscle tissue and IntraMAT, the following were classified as individual muscle groups: quadriceps femoris (QF) (i.e., sum of the rectus femoris [RF], vastus lateralis [VL], vastus intermedius [VI], and vastus medialis [VM]); hamstrings (HM) (i.e., sum of the biceps femoris short head [BFs], biceps femoris long head [BFl], semitendinosus [ST], and semimembranosus [SM]; and hip adductors (AD) (i.e., sum of adductor longus [AL], adductor brevis [AB], adductor magnus [AM], sartorius [Sar], and gracilis [Gr]). Furthermore, the sum of the muscle tissue CSA of the QF, HM, and AD was calculated as in that of the whole thigh.

InterMAT CSA was calculated using the following equation:

$$\;\text{InterMAT}\;\text{CSA}\left({\text{cm}}^{2}\right)=\left(\text{InterMAT}\;\text{pixel}\;\text{number}\right)\times {\left(\text{FOV}/\text{matrix}\;\text{size}\right)}^{2}$$

SAT CSA was calculated using the following equation:

$$\;\text{SAT}\;\text{CSA}\left({\text{cm}}^{2}\right)=\text{total thigh CSA}‐\left(\begin{array}{c} \text{muscle tissue CSA}+\text{IntraMAT CSA}\\ +\text{InterMAT CSA}+\text{femur CSA} \end{array}\right)$$

Finally, the volumes of the muscle tissue, IntraMAT, InterMAT, and SAT, respectively, were determined using the following equations (Nordez et al., 2009):

$$\text{Muscle}\;\text{tissue}\;\text{volume}\left({\text{cm}}^{3}\right)=\sum_{n}e_{i}\times \text{Muscle}\;\text{tissue}\;{\text{CSA}}_{i}$$

$$\;\text{IntraMAT}\;\text{volume}\left({\text{cm}}^{3}\right)=\sum_{n}e_{i}\times \text{IntraMAT}\;{\text{CSA}}_{i}$$

$$\;\text{Inter}\;\text{MAT}\;\text{volume}\left({\text{cm}}^{3}\right)=\sum_{n}e_{i}\times \text{InterMAT}\;{\text{CSA}}_{i}$$

$$\text{SAT}\;\text{volume}\left({\text{cm}}^{3}\right)=\sum_{n}e_{i}\times \text{SAT}\;{\text{CSA}}_{i}$$

in which *n* was the number of slices used and *e_i_* was the known distance between slices.

IntraMAT content was determined using the following equations (Akima et al., 2015):

$$\text{IntraMAT content}\left(\%\right)=\left\{\text{IntraMAT volume}/(\text{muscle tissue volume}+\text{IntraMAT volume})\right\}\times \text{100}$$

### 2.4 Bed rest procedures

The participants attended the facility for baseline data collection 8 or 9 days before starting the 6° head‐down tilt bed rest for 60 days. They remained in this position throughout the bed rest period except for the RE and VRE groups during exercise sessions. Weight‐bearing postures were not permitted, and any physical activities were restricted to a minimum in the CTR group. During bed rest, the participants performed all hygiene activities in the bed rest position. Daily meals during bed rest were provided according to the German Nutrition Society guidelines (50% to 55% carbohydrate, 30% to 35% fat, and 15% to 20% protein). The participants were then advised that food could not be exchanged with other participants, meals were to be consumed completely, and no snacks were allowed between meals. This procedure was essentially the same as that in previous study (Belavý et al., 2010).

### 2.5 Resistance exercise intervention

This bed rest study involved the evaluation of two different exercise approaches. Overall, the lower quadrant regions of the body were targeted with exercise (3 days per week) during bed rest. The VRE group performed the same resistance exercises as the RE group but with the addition of simultaneous whole‐body vibration (applied at the feet) during high‐load resistance exercises. In a given exercise session, the following exercises were performed:

1. Warm‐up: bilateral squat exercises (from 10° to 90° knee flexion and back to the starting position of 10° knee flexion) with 50% of maximal force for 64 s. Participants performed both the concentric and eccentric phases of the exercise for 4 s each. Eight continuous (i.e., without pause) repetitions were performed. In the VRE group, the vibration was set to 24 Hz (amplitude 3.5–4.0 mm). A 2 min break was given after the warm‐up.
2. Bilateral squats (from 10° to 90° knee flexion and back with the concentric and eccentric phases for 4 s each) were performed during the first training session at 75% and after the second training session at 80% at the most; the participants performed the exercise continuously until exhaustion. From the third training session, the force level was increased by 5% at each session until the participant could only perform eight repetitions. In the subsequent sessions, if the participant improved in such a way that they could perform more than 10 repetitions in two adjoining sessions, the force level was increased by 5% again. If a participant could not successfully complete six repetitions in two successive sessions, then the force was decreased by 5%.

In the VRE group, the vibration frequency progressed from 20 to 24 Hz (amplitude: 3.5 to 4.0 mm) from the first to the third training and was then maintained at this level for the rest of the study. A 5 min break was then given after the exercise.

- Single‐leg heel raises were performed on the left and right legs from a maximal plantarflexion to a maximal dorsiflexion against a force equivalent to approximately 1.3 times their first training session body weight. The forefoot was positioned on the bottom of the force plate with the heel hanging over the edge. The participants were asked to perform the movement as quickly as possible while moving from a full plantar flexion to a full dorsiflexion. Typically, a movement frequency of 0.4 to 0.7 Hz was achieved. Furthermore, the participant was instructed to hold the knee at full extension to avoid assistance via the knee musculature. The exercise was performed until exhaustion (i.e., when the participant could no longer perform the movement accurately).

For the VRE group, the vibration frequency was set to 26 Hz (amplitude 3.5 to 4.0 mm). Upon completion of the exercise for the first leg, a 90 s break was given before the commencement of the exercise for the other leg, and that for both legs, a 4 min break. If the participant could not perform the exercise for more than 30 s, then the load was reduced by 5%. If the participant was able to perform the exercise for more than 50 s, the load was increased by 5%.

- Double‐leg heel raises were performed in the same manner as for the single‐leg heel raises except that the resistive force was set to approximately 1.8 times body weight. The exercise was performed until exhaustion.

For the VRE group, the vibration frequency was set to 26 Hz (amplitude 3.5 to 4.0 mm). A 2 min break was given after the completion of the exercise. If the participant could not perform the exercise for more than 40 s, then the load was reduced by 5%. If the participant was able to perform the exercise for more than 55 s, the load was then increased by 5%.

- With their feet positioned on the platform, the participants extended their hips and lumbar spine, dorsiflexed their ankles, and maintained their knees at full extension. A plank‐like body lift was performed. Participants were required to maintain this position for 60 s. During the exercise, a force 1.5 times body weight was applied at the shoulders. Vibration frequency was set to 16 Hz (amplitude: 3.5–4.0 mm) in the VRE group. Furthermore, the exercise was not progressed.

The actual loading time was 5 to 6 min per exercise session, and the total exercise time was 22 min, including rest periods.

### 2.6 Reproducibility analysis

The manual segmentation of the mid‐thigh muscle compartments was repeated twice in 10 randomly selected participants by the researcher to evaluate the intra‐observer reproducibility of the segmentation process. The intraclass correlation coefficients (ICC 2.1) for IntraMAT and InterMAT were 0.892 and 0.970, respectively (both *p* < .001). The standard errors of measurement representing the absolute consistency were 0.2–1.7 cm^2^．

### 2.7 Statistical analysis

All continuous variables were expressed as the mean ± standard deviation. The change before and after the intervention was calculated; a within‐group design was used with a 95% CI. Muscle tissue, IntraMAT, InterMAT, and SAT volumes were analyzed using a two‐factor repeated measures analysis of variance (ANOVA): [time (before and after) × group (RE group and VRE group)] and [time (before and after) × group (CTR group and RE group plus VRE group; TR group)]. Significant main effects and interactions were compared using the Bonferroni post‐hoc test. Additionally, the Aspin–Welch *t*‐test was used to determine the differences between the percentage change in the CTR and TR groups. Pearson's correlation was used to determine the relation between the change in the muscle tissue, IntraMAT, and InterMAT volumes before and after the intervention. We also examined the relation between the percentage changes in IntraMAT, InterMAT, and SAT using Pearson's correlation. Statistical significance was set to *p *< .05. As for the indices of the effect size, Cohen's *d* (for a post‐hoc test) and partial *η* ^2^ (for ANOVA) were also calculated. Furthermore, the correlation power analysis (for a post‐hoc test) was performed using the effect size (*r*).

## 3 RESULTS

In the RE and VRE groups, no significant main effects of time (*p* = .317 and 0.844; *F* _(1,11)_ = 0.041 and 1.101; and partial *η* ^2^ = 0.004 and 0.615, respectively) were observed concerning the volumes of muscle tissue, InterMAT, and SAT. By contrast, the IntraMAT volume showed a significant main effect of time (*p* = .002; *F*_(1,11)_ = 40.58; partial *η* ^2^ = 0.615). Furthermore, the volumes of muscle tissue and IntraMAT showed no significant main effect of group (*p* = .110 and 0.260; *F*_(1,11)_ = 3.014 and 1.412; and partial *η* ^2^ = 0.215 and 0.044, respectively), whereas the volumes of the InterMAT and SAT showed significant main effect of group (*p* = .021 and 0.013; *F*_(1,11)_ = 7.262 and 8.685; and partial *η* ^2^ = 0.398 and 0.441, respectively). However, no significant time‐by‐group interaction in the volumes of the muscle tissue, IntraMAT, InterMAT, and SAT was observed (*p* = .350 to 0.719; *F*_(1,11)_ = 0.438 to 1.412; partial *η* ^2^ = 0.012 to 0.080). Therefore, we considered the RE and VRE groups interventions to have had equivalent effects on the muscle and adipose tissues. Accordingly, the two groups were pooled as the resistance exercise intervention group (TR group).

Table 2 shows the muscle tissue volume of the individual muscles of the thigh before and after the bed rest. Furthermore, the muscle tissue volumes of the RF, VL, VI, VM, AL, AM, QF, AD, and whole thigh showed significant time‐by‐group interaction (*p* = .001 to 0.026; *F* _(1,18)_ = 5.21 to 23.01; partial *η* ^2^ = 0.225 to 0.572). The muscle tissue volumes of BFl and ST within HM showed a significant main effect of time (*p* = .001 to 0.010; *F* _(1,18)_ = 40.58 to 75.27; partial *η^2^* = 0.693 to 0.807) and no significant time‐by‐group interaction and main effect of group.

**TABLE 2:** Muscle tissue volume of the individual thigh muscles for the resistance exercise intervention (TR) and control (CTR) groups

	TR group			CTR group		
	Means ± *SD*		From before to after mean (95%CI)	Means ± *SD*		From before to after mean (95%CI)
	Before (cm^3^)	After (cm^3^)	Within‐group change (cm^3^)	Before (cm^3^)	After (cm^3^)	Within‐group change (cm^3^)
RF	283.1 ± 50.8	285.2 ± 45.5	2.1 (−6.4, 10.7)	305.0 ± 48.8	285.6 ± 51.0	−19.4 (−43.6, 4.8)
VL	684.6 ± 161.4	718.8 ± 135.7	34.2 (−20.9, 89.3)	719.9 ± 158.3	574.6 ± 132.9*	−145.3 (−208.7, −81.9)
VI	515.4 ± 82.1	509.4 ± 87.4	−6.0 (−32.1, 20.2)	524.4 ± 62.7	417.0 ± 75.5*	−107.5 (−154.0, −60.9)
VM	510.8 ± 99.9	517.3 ± 113.1	6.5 (−26.9, 39.9)	562.3 ± 55.5	492.2 ± 63.5*	−70.1 (−122.5, −17.6)
QF	1993.9 ± 361.5	2030.7 ± 346.1	36.9 (−64.4, 138.2)	2,111.6 ± 182.2	1769.4 ± 211.7*	−342.2 (−499.6, −184.8)
BFs	109.7 ± 23.0	108.9 ± 25.2	−0.8 (−8.1, 6.4)	113.6 ± 20.8	113.6 ± 29.0	0.0 (−19.3, 19.3)
BFl	210.0 ± 42.3	183.0 ± 35.5	−27.2 (−37.7, −16.7)	228.3 ± 20.6	196.2 ± 30.6	−19.2 (−51.9, −12.3)
ST	190.4 ± 46.1	189.6 ± 44.3	−0.8 (−10.0, 8.3)	201.2 ± 38.6	197.1 ± 30.6	−4.1 (−17.6, 9.4)
SM	241.5 ± 50.9	211.0 ± 48.2	−30.5 (−41.5, −19.6)	272.6 ± 34.9	235.9 ± 37.1	−36.7 (−48.4, −25.0)
HM	751.8 ± 133.7	692.4 ± 133.7	−59.4 (−90.1, −28.6)	815.6 ± 66.7	742.7 ± 80.0	−72.9 (−97.5, −48.3)
AL	174.3 ± 41.4	178.0 ± 34.8	3.6 (−6.4, 13.7)	182.1 ± 26.0	166.0 ± 30.5	−15.6 (−31.5, 0.24)
AM	570.6 ± 108.6	577.8 ± 98.3	1.6 (−21.5, 24.6)	525.6 ± 56.6	482.3 ± 46.1	−50.8 (−111.2, 9.7)
AB	119.2 ± 34.9	118.3 ± 32.5	−0.9 (−10.7, 8.8)	133.9 ± 13.0	140.4 ± 24.5	0.4 (−10.3, 11.1)
Sar	129.2 ± 40.2	129.8 ± 37.0	0.6 (−6.1, 7.3)	134.0 ± 23.4	131.8 ± 30.3	−2.2 (−11.5, 7.2)
Gr	103.8 ± 24.3	107.2 ± 21.6	3.4 (−1.1, 8.0)	95.7 ± 18.0	99.6 ± 21.1	3.9 (−2.1, 9.8)
AD	1,067.6 ± 196.0	1,075.9 ± 182.8	8.3 (−26.4, 42.9)	1,071.2 ± 59.5	1,020.1 ± 75.1	−64.3 (−137.2, 8.5)
Whole thigh	3,780.0 ± 696.2	3,796.1 ± 643.4	16.6 (−84.5, 117.7)	3,998.5 ± 190.5	3,532.7 ± 311.8*	137.3 (74.9, 199.7)

RF, rectus femoris; VL, vastus lateralis; VI, vastus intermedius; VM, vastus medialis; QF, quadriceps femoris; BFs, biceps femoris, short head; BFl, biceps femoris, long head; ST, semitendinosus; SM, semimembranosus; HM, hamstrings; AL, adductor longus; AM, adductor magnus; AB, adductor brevis; Sar, sartorius; Gr, gracilis; AD, hip adductors; * *p* < .05, versus Before.

In the CTR group, a post‐hoc test revealed that the muscle tissue volumes of the VL, VI, VM, QF, and whole thigh significantly decreased after bed rest (*p* = .001, 0.001, 0.017, 0.002, and 0.004, respectively). Furthermore, the muscle tissue volume of all muscles were maintained in the TR group.

**FIGURE 1:**
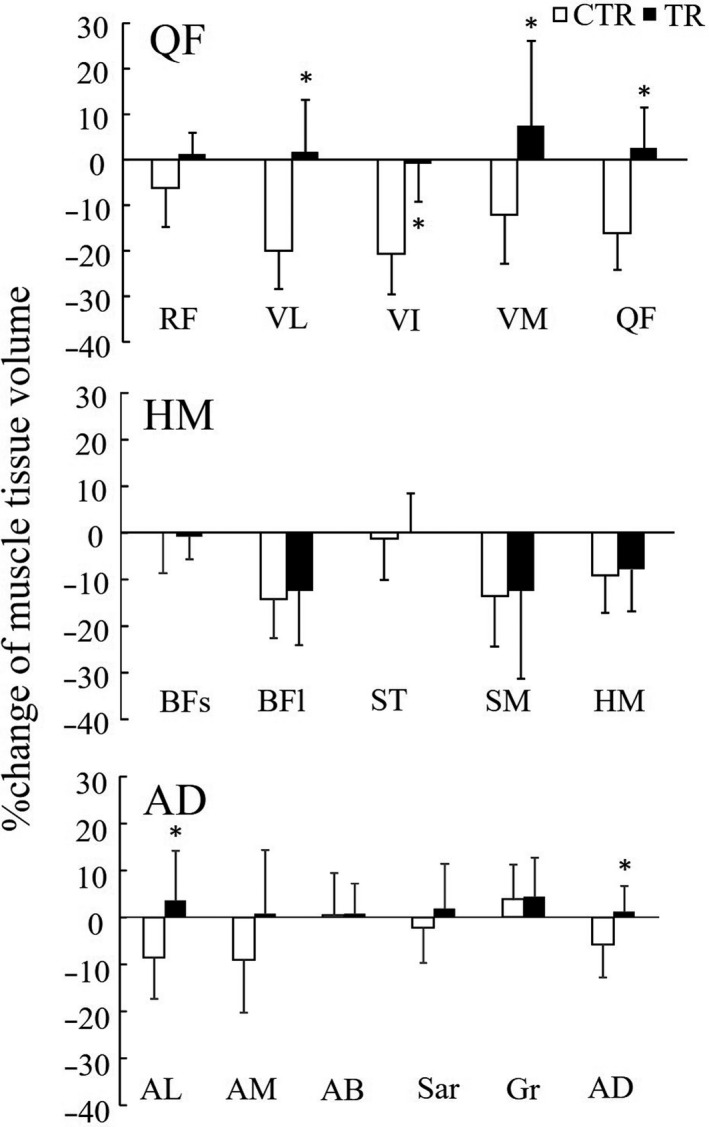
Muscle tissue volume percentage change in individual muscles. *CTR* control group, *TR* resistance exercise intervention group, *RF* rectus femoris, *VL* vastus lateralis, *VI* vastus intermedius, *VM* vastus medialis, *QF* quadriceps femoris, *BFs* biceps femoris, short head, *BFl* biceps femoris, long head, *ST* semitendinosus, *SM* semimembranosus, *HM* hamstrings, *AL* adductor longus, *AM* adductor magnus, *AB* adductor brevis, *Sar* sartorius, *Gr* gracilis, *AD* hip adductors, *p < .05, versus CTR

The percentage changes in the muscle tissue volume for the CTR and TR groups are shown in Figure 1. In the CTR group, the percentage changes in the muscle tissue volumes of the VM, VI, VL, AL, QF, and AD were significantly lower than that in the TR group (*p < *.001, *d* = 1.220 to 2.310; Figure 1).

Table 3 shows the IntraMAT volume of the thigh muscle groups before and after the bed rest. No time‐by‐group interactions in the individual muscle groups were observed; however, a significant main effect of time was observed (*p* = .001 to 0.020; *F* _(1,18)_ = 7.54 to 28.20; partial *η^2^* = 0.214 to 0.747) in the IntraMAT volumes of the RF, VI, VL, BFs, BFl, SM, AL, AM, Sar, Gr, QF, HM, AD, and whole thigh (Table 3). Furthermore, a significant main effect of group was observed in the IntraMAT volumes of the VM, AB, and AM (*p* = .001 to 0.040; *F* _(1,18)_ = 6.87 to 19.50; partial *η^2^* = 0.389 to 0.765).

**TABLE 3:** Intramuscular adipose tissue volume of the individual thigh muscles for the resistance exercise intervention (TR) and control (CTR) groups

	TR group			CTR group		
	Means ± *SD*		From before to after mean (95%CI)	Means ± *SD*		From before to after mean (95%CI)
	Before (cm^3^)	After (cm^3^)	Within‐group change (cm^3^)	Before (cm^3^)	After (cm^3^)	Within‐group change (cm^3^)
RF	36.5 ± 7.5	30.3 ± 5.2	−6.1 (−9.9, −2.3)	40.8 ± 7.9	38.0 ± 9.2	−2.8 (−5.3, −0.3)
VL	144.4 ± 45.5	133.4 ± 51.4	−11.0 (−23.1, 1.0)	133.4 ± 16.3	107.0 ± 16.8	−26.4 (−46.2, −6.6)
VI	102.7 ± 26.6	94.8 ± 33.5	−7.9 (−18.1, 2.3)	109.6 ± 27.7	109.0 ± 29.2	−13.0 (−26.9, 0.8)
VM	110.1 ± 36.5	93.4 ± 32.8	−16.7 (−32.1, −1.2)	103.7 ± 23.0	96.6 ± 26.4	−6.0 (−16.7, 4.6)
QF	393.7 ± 106.4	351.9 ± 111.9	−41.7 (−65.8, −17.6)	387.5 ± 51.2	344.0 ± 71.8	−48.2 (−79.8, −16.8)
BFs	25.6 ± 4.7	21.8 ± 6.9	−3.8 (−6.0, −1.6)	27.6 ± 8.3	24.1 ± 8.9	−3.5 (−10.0, 3.1)
BFl	49.3 ± 21.5	42.6 ± 23.2	−6.7 (−12.3, −1.1)	51.9 ± 12.3	41.6 ± 15.4	−10.3 (−24.7, 4.1)
ST	37.4 ± 16.6	27.2 ± 10.9	−10.2 (−14.6, −5.9)	48.3 ± 16.0	36.4 ± 9.6	−11.8 (−26.1, 2.3)
SM	58.1 ± 19.5	55.0 ± 21.6	−3.1 (−9.3, 3.0)	55.2 ± 12.7	54.7 ± 15.8	−0.5 (−18.7, 17.6)
HM	170.5 ± 55.3	146.6 ± 56.3	−23.9 (−36.4, −11.3)	183.0 ± 38.0	156.9 ± 44.2	−26.2 (−72.7, 20.3)
AL	24.8 ± 7.3	20.4 ± 8.1	−4.4 (−6.8, −2.0)	29.7 ± 5.1	27.2 ± 6.8	−2.7 (−6.3, 0.8)
AM	128.0 ± 50.0	112.9 ± 46.3	−15.1 (−25.3, −5.0)	126.9 ± 16.5	101.0 ± 23.5	−22.3 (−39.6, −5.2)
AB	16.0 ± 5.5	13.2 ± 4.7	−2.8 (−4.7, −0.9)	28.7 ± 6.9	28.6 ± 18.5	0.4 (−3.2, 4.0)
Sar	44.3 ± 12.5	39.6 ± 13.7	−4.7 (−8.4, −1.0)	53.9 ± 17.4	47.9 ± 19.1	−6.0 (−13.3, 1.3)
Gr	22.0 ± 7.9	19.0 ± 7.5	−3.0 (−5.3, −0.6)	24.9 ± 5.1	20.8 ± 4.1	−4.1 (−7.2, −0.9)
AD	235.2 ± 70.7	205.2 ± 68.7	−30.0 (−43.8, −16.3)	264.1 ± 32.3	215.3 ± 49.8	−41.4 (−62.3, −20.5)
Whole thigh	795.5 ± 225.1	703.4 ± 233.3	−92.1 (−139.4, −44.7)	834.6 ± 67.3	721.7 ± 161.7	−111.2 (−196.0, −26.3)

RF, rectus femoris; VL, vastus lateralis; VI, vastus intermedius; VM, vastus medialis; QF, quadriceps femoris; BFs, biceps femoris, short head; BFl, biceps femoris, long head; ST, semitendinosus; SM, semimembranosus; HM, hamstrings; AL, adductor longus; AM, adductor magnus; AB, adductor brevis; Sar, sartorius; Gr, gracilis; AD, hip adductors; * *p*< .05, versus Before.

**FIGURE 2:**
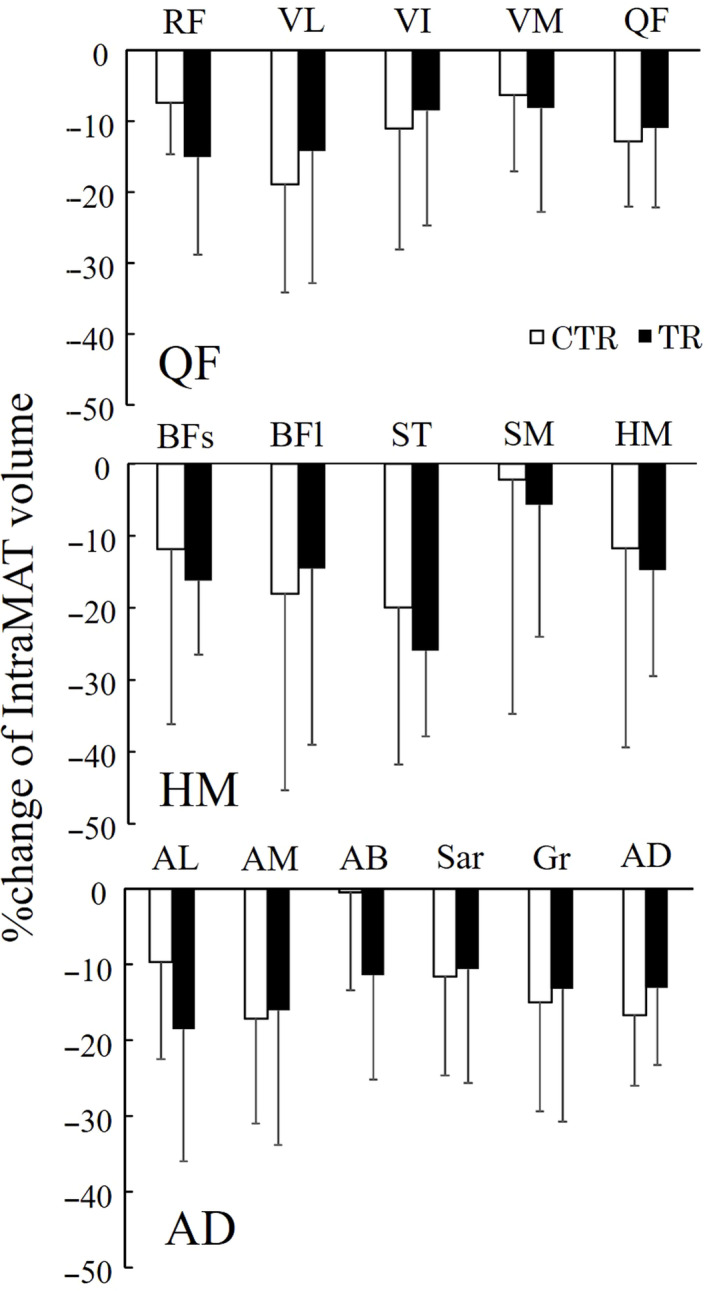
Intramuscular adipose tissue (IntraMAT) volume percentage change in individual muscles. *CTR* control group, *TR* resistance exercise intervention group, *RF* rectus femoris, *VL* vastus lateralis, *VI* vastus intermedius, *VM* vastus medialis, *QF* quadriceps femoris, *BFs* biceps femoris, short head, *BFl* biceps femoris, long head, *ST* semitendinosus, *SM* semimembranosus, *HM* hamstrings, *AL* adductor longus, *AM* adductor magnus, *AB* adductor brevis, *Sar* sartorius, *Gr* gracilis, *AD* hip adductors

The percentage changes in the IntraMAT volumes for the CTR and TR groups are shown in Figure 2. No significant difference in the percentage change of the IntraMAT volume in the CTR and TR groups was observed (Figure 2).

Table 4 shows the IntraMAT content of the thigh muscle groups. The IntraMAT content in the whole thigh and individual muscle groups did not show any time‐by‐group interactions or main effect of group; however, a significant main effect of time in QF and AD was observed (*p* = .041 and 0.001; *F* _(1,18)_ = 4.84 and 17.22; partial *η^2^* = 0.212 and 0.489, respectively).

**TABLE 4:** Intramuscular adipose tissue content of the thigh muscle groups for the resistance exercise intervention (TR) and control (CTR) groups

	TR group			CTR group		
	Means ± *SD*		From before to after mean (95%CI)	Means ± *SD*		From before to after mean (95%CI)
	Before (%)	After (%)	Within‐group change	Before (%)	After (%)	Within‐group change
QF	16.2 ± 3.0	14.6 ± 3.2	−1.6 (−3.2, 0.0)	15.6 ± 2.2	16.0 ± 3.2	0.4 (−0.9, 1.7)
HM	17.9 ± 3.6	17.1 ± 4.4	−0.6 (−2.6, 1.3)	18.3 ± 3.3	17.5 ± 4.4	−0.8 (−5.3, 3.6)
AD	18.1 ± 3.7	15.9 ± 3.5	−2.2 (−3.5, −1.0)	19.2 ± 1.4	17.4 ± 3.4	−1.8 (−3.8, 0.1)
Whole thigh	17.1 ± 3.1	15.4 ± 3.3	−2.1 (−3.7, −0.5)	17.3 ± 1.1	17.0 ± 3.7	−0.3 (−2.4, 1.8)

QF, quadriceps femoris; HM, hamstrings; AD, hip adductors.

Table 5 shows the volumes of the SAT and InterMAT before and after the bed rest. Significant time‐by‐group interaction in the volume of SAT was observed (*p* = .010; *F* _(1,18)_ = 18.63; partial *η* ^2^ = 0.509) with no significant main effects of time and group. However, no significant main effects of time, group, and time‐by‐group interaction were observed in the InterMAT volume (Table 5). Furthermore, a post‐hoc test revealed significant increased in SAT in the CTR group andno change in the TR group after bed rest (*p* = .002; Table 5).

**TABLE 5:** Subcutaneous adipose tissue (SAT) and intermuscular adipose tissue (InterMAT) volumes of the thigh for the resistance exercise intervention (TR) and control (CTR) groups

	TR group			CTR group			
	Mean ± *SD*		From before to after mean (95%Cl)	Mean ± *SD*			From before to after mean (95%Cl)
	Before (cm^3^)	After (cm^3^)	Within‐group change (cm^3^)	Before (cm^3^)	After (cm^3^)	Within‐group change (cm^3^)	
SAT	1,217.6 ± 613.1	1,244.8 ± 571.7	27.2 (−25.6, 80.0)	1,190.3 ± 253.6	1,327.6 ± 233.0*	137.3 (74.9, 199.7)	
InterMAT	427.4 ± 219.3	432.2 ± 206.8	4.8 (−35.4, 44.9)	375.0 ± 128.4	355.5 ± 182.4	15.3 (−43.3, 73.8)	

* *P* < .05, versus Before.

**FIGURE 3:**
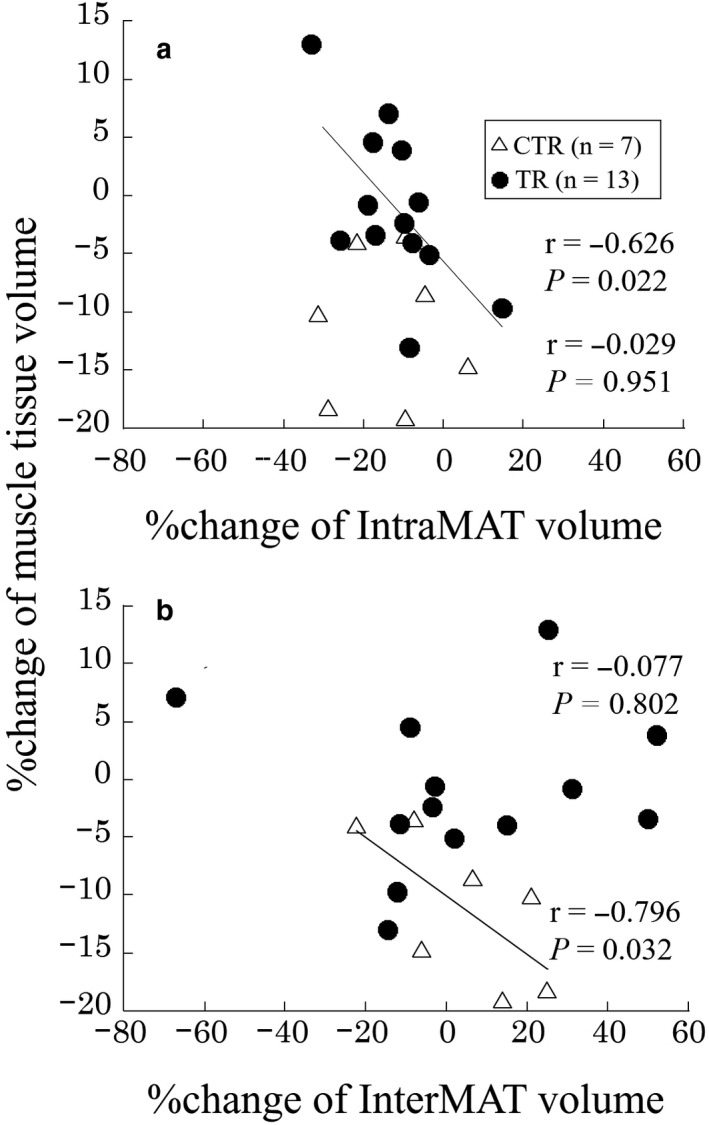
Relation between the percentage changes of muscle tissue, intramuscular adipose tissue (IntraMAT), and intermuscular adipose tissue (InterMAT) volumes. (A) Muscle tissue volume percentage change and IntraMAT volume percentage change. (B) Muscle tissue volume percentage change and InterMAT volume percentage change. *CTR* control group, *TR* resistance exercise intervention group

**FIGURE 4:**
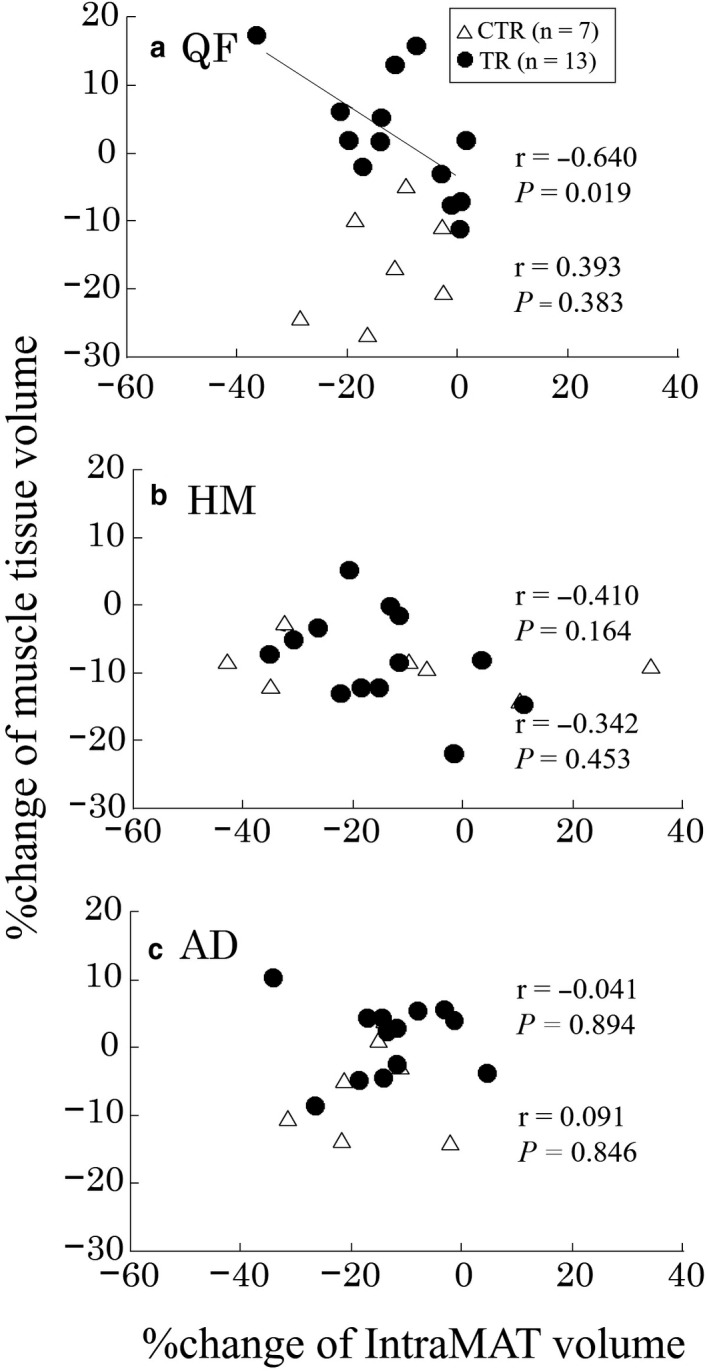
Relation between the percentage changes of muscle tissue volume and intramuscular adipose tissue (IntraMAT) volume in the thigh muscle groups. (a) *QF* quadriceps femoris, (b) *HM* hamstrings, (c) *AD* hip adductors. *CTR* control group, *TR* resistance exercise intervention group

The relation between the percentage changes in the muscle tissue, IntraMAT and InterMAT volumes are shown in Figures 3 and 4. The percentage changes of the IntraMAT volumes (95%CI, −19.1 to − 5.2) were significantly correlated with those of the muscle tissue volumes (95%CI, −2.2 to 3.8) in the TR group (*r* = −.626, *p* = .022; Figure 3a), but not in the CTR group (*r* = −.029, n.s.). The power analysis result was medium (1‐β = 0.677, *α* = .05) in the TR group. The percentage change of the InterMAT volume (95%CI, −14.0 to 19.7) was a significantly correlated with that of the muscle tissue volume (95%CI, −17.1 to −5.2) in the CTR group (*r* = −.796, *p *= .032; Figure 3b), but not in the TR group (*r* = −.077, n.s.). In the QF, the percentage change of the IntraMAT volume (95%CI, −17.7 to −4.3) was a significantly correlated with that of the muscle tissue volume (95%CI, −3.0 to 7.9) in the TR group (*r* = −.640, *p* = .019; Figure 4a), whereas no significant relation was observed in the CTR group (*r* = .393, n.s.). The power analysis result was medium (1‐β = 0.659, α = 0.05) in the TR group. Furthermore, no significant HM and AD relations were observed in either the CTR or TR group (Figure 4b and 4c).

A relation between the percentage change in the volumes of the IntraMAT, InterMAT, and SAT was observed. However, no significant relation was observed between the percentage change of IntraMAT volume and that of InterMAT volume (*r* = −.247, n.s.). We also observed no significant relation for the rest of the combinations, that is, IntraMAT volume and SAT volume (*r* = −.400, n.s.) or InterMAT volume and SAT volume (*r* = −.024, n.s.).

## 4 DISCUSSION

We investigated the effect of 8 weeks of bed rest with or without resistance exercise interventions on the volumes of muscle tissue and adipose tissues (IntraMAT, InterMAT, and SAT) of the thigh. The volumes of the muscle tissue, InterMAT, and SAT were maintained by resistance exercise interventions during bed rest. However, IntraMAT volumes were significantly decreased in both the CTR and TR groups. This implies that during 8 weeks of bed rest, the adipose tissues adaptation differed depending on the location. Importantly, the present study showed that the relative changes in muscle tissue volume were negatively correlated with the IntraMAT volume in the TR group and InterMAT volume in the CTR group. These findings indicate that the change in muscle tissue size related to bed rest and exercise interventions were related to the amount of adipose tissue within/between the muscle, and we provide unique evidence of interactions between muscle tissue and adipose tissue in humans.

We then investigated the effect of 8 weeks of bed rest with resistance exercise interventions on the volumes of the muscle and adipose tissues using an established segmentation technique (Akima et al., 2015, 2016; Manini et al., 2007; Ogawa et al., 2017; Yoshiko et al., 2018). After bed rest, the muscle tissue volume of the QF in the TR group was maintained; however, that in the CTR group a significantly decreased (Figure 1 and Table 2). Interestingly, significant reductions in muscle tissue volume were found in the HM for both the CTR and TR groups as a result of bed rest. A similar result was reported in the study of Akima et al. (2000), wherein the muscle size of the QF was maintained, but not HM, because of the “isometric” leg press training during 20 days of bed rest (Akima et al., 2000). Akima et al. (2001) also reported that the muscle sizes of both the QF and the HM were maintained because of the “dynamic” leg press training during 20 days of bed rest (Akima et al., 2001). In the present study, contrary to the previous studies, significant reduction in the HM muscle tissue volume was observed in the TR group even though the participants were involved in several different types of resistance exercise interventions, including dynamic leg press, during bed rest (Table 2). Additionally, no significant intergroup difference was observed (Figure 1), and the reason for this is unclear. The difference could be associated with the longer duration of bed rest and variation of the individual data on muscle tissue volume. Adaptation of the HM is also likely to be susceptible to this model of disuse. During the same period, i.e., 20 days, of unloading experiments, HM muscle size significantly decreased after the bed rest model (9.6%); however, no significant reduction was observed after the unilateral lower limb unloading model (1.3%) (Sato et al., 2010). Furthermore, posture during resistance exercise interventions, e.g., knee and/or hip joint angle, tends to influence the HM muscle size.

Significant reductions in the IntraMAT volume of the CTR and TR groups after bed rest were observed, which was unexpected. A conflicting result had been reported by Manini et al. (2007), which showed that significant increases in the sum IntraMAT and InterMAT were observed in the thigh and calf muscles after 28 days of unilateral lower limb unloading (Manini et al., 2007). The crucial difference between the present study and that of Manini et al. (2007) is that we strictly controlled the calorie intake for the participants during the bed rest period, which was not the case in their study. In the study by Manini et al. (2007), no description regarding the calorie intake was included; therefore, it appears that the calorie intake was not controlled during the experiment. Rudiwill et al. (2018) showed increases in the sum IntraMAT and InterMAT contents in the calf when adjustments were made to the calorie intake during 21 days of bed rest (Rudwill et al., 2018). Conversely, no significant changes in the sum IntraMAT and InterMAT volumes after a week of bed rest were found (Dirks et al., 2016). Therefore, IntraMAT and InterMAT changes induced by bed rest were likely to be small, and the relative increase in the IntraMAT and InterMAT volumes could be due to the decreasing muscle tissue volume rather than an increase in the adipose tissue volume itself. A previous study reported that the sum of IntraMAT and InterMAT CSAs decreased by approximately 5% after 28 days of bed rest, with resistance exerciseinterventions performed six times per week (Brooks et al., 2014). Overall, both the balance of the calorie intake and exercise‐induced calorie consumption could be closely related to the reductions in the IntraMAT and InterMAT volumes in the present study.

Another intriguing result was that bed rest‐induced changes were not similar between the IntraMAT and SAT volumes in the CTR group. A previous study had reported that the sum of IntraMAT and InterMAT contents in the calf increased, whereas the SAT volume of the trunk decreased after 21 days of bed rest (Rudwill et al., 2018). Additionally, this evidence proposes that the adipose tissue could be fixed site by site following its accumulation (Rudwill et al., 2018). This could be regulated by factors such as hormones, cytokines, enzymes, and/or the type of muscle fiber (Nagarajan et al., 2017; Schoettl et al., 2018). In the present study, we could not identify which factor was the major regulator.

We found that the percentage change in muscle tissue volume was significantly correlated with that of the IntraMAT volume in the TR group (Figure 3). This finding explains interaction between muscle tissue and adipose tissue that accumulate in various locations of the thigh. IntraMAT is defined as the adipose tissue infiltration within the muscle, whereas the InterMAT is defined as the adipose tissue of the subfascia between the muscles (Shen et al., 2003; Yoshiko et al., 2018). The physiological meaning of the different types of adipose tissue deposition patterns is unclear. Both the IntraMAT and InterMAT are known to be related to insulin resistance (Boettcher et al., 2009; Goodpaster et al., 2000). Although the IntraMAT and InterMAT are considered to be types of adipose tissue similar to the visceral adipose tissue, they each have different adaptations as a result of disuse (Yoshiko et al., 2018). Yoshiko et al. (2018) found a significant negative correlation between the percentage change of the muscle tissue CSA and that of the IntraMAT CSA after 4 weeks of cast immobilization in patients with orthopedic problems in the foot (Yoshiko et al., 2018). Conversely, we found that the percentage change of muscle tissue volume in the whole thigh was a significantly correlated only with that of the InterMAT volume in the CTR group (Figure 3b), but not with that of the IntraMAT volume (Figure 3a). This could be due to a different disuse model and the participants’ physical characteristics. Additionally, the power analysis results in the present study were medium (1‐β = 0.677 and 0.659). Therefore, we need further studies with a larger population to verify our findings. Furthermore, the correlation analysis revealed an inverse relation between the percentage change of the muscle tissue volume and that of the IntraMAT volume of the QF in the TR group (*r* = −.640, *p* = .019), but not in the CTR group (Figure 4a). The primary reason for this relation is because this muscle group was targeted by the resistance exercises, such as bilateral squats. Additionally, resistance training‐induced adaptation was primarily observed in QF (Table 2 and Figure 1). This result clearly showed that a greater reduction in the percentage change of the IntraMAT volume represents a greater increase in that of the muscle tissue volume. Previous cross‐sectional and longitudinal studies has reported thesignificant relation between the muscle tissue size and IntraMAT size (Akima et al., 2015; Yoshiko et al., 2018). In the cross‐sectional study by Akima et al. (2015) a significant negative relationbetween muscle tissue size and IntraMAT content in both older and younger individuals were observed (*r* = .672 to .733) (Akima et al., 2015). Similarly, an inverse significant relation between muscle tissue size and IntraMAT size (ρ = −0.86, *p* < .01) in patients with post‐fracture non‐weight‐bearing immobilization for 4 weeks was observed (Yoshiko et al., 2018). Furthermore, the results of the present study are consistent with the aforementioned studies, which imply that muscle tissue and IntraMAT can interact with each other to affect their size.

A few reasons could explain the significant relation we observed between the percentage change of muscle tissue volume and that of the IntraMAT volume of the QF in the TR group and not in the CTR group (Figure 4a).The IntraMAT represents the adipose tissues located within the muscle. Specifically, the IntraMAT is referred to as the adipose tissues in the interstitium, that is, extramyocellular lipids (Akima et al., 2016), considering the amount of lipids outside the muscle fibers, which is approximately 5‐ to 10‐fold greater than that within the muscle fibers. A previous study suggested that when muscle atrophy was induced as a result of disuse, the extramyocellular space became larger and that IntraMAT could quickly fill the space left by the atrophied muscle tissue (Yoshiko et al., 2018). The present study also suggests that resistance training‐induced muscle hypertrophy could decrease IntraMAT size, indicating that the IntraMAT has greater adaptive plasticity.

In conclusion, we observed thatresistance exerciseintervention during 8 weeks of bed rest prevented muscle atrophyand SATincrease in the thigh. However, changes in the IntraMAT and InterMAT volumes were not different in the CTR and TR groups. These results demonstrate that during 8 weeks of bed rest adipose tissue adaptation differs depending on location.Additionally, the change in muscle tissue size was inversely correlated with that of IntraMAT size in the TR group and that of InterMAT size in the CTR group; however, further study with a larger population is required to explain those relationships.

## CONFLICT OF INTEREST

The authors declare no conflicts of interest associated with this manuscript.
